# Supplementary material for: Predominance of Calcium Pyrophosphate Crystals in Synovial Fluid Samples of Patients at a Large Tertiary Center
Source: Diagnostics (Basel). 2025 Apr 1;15(7):907. doi: 10.3390/diagnostics15070907 (PMC11988333; doi:10.3390/diagnostics15070907)
Supplement: Supplementary file 1 [file diagnostics-15-00907-s001.zip › diagnostics-3526147-supplementary.pdf]

diagnostics

MDPI

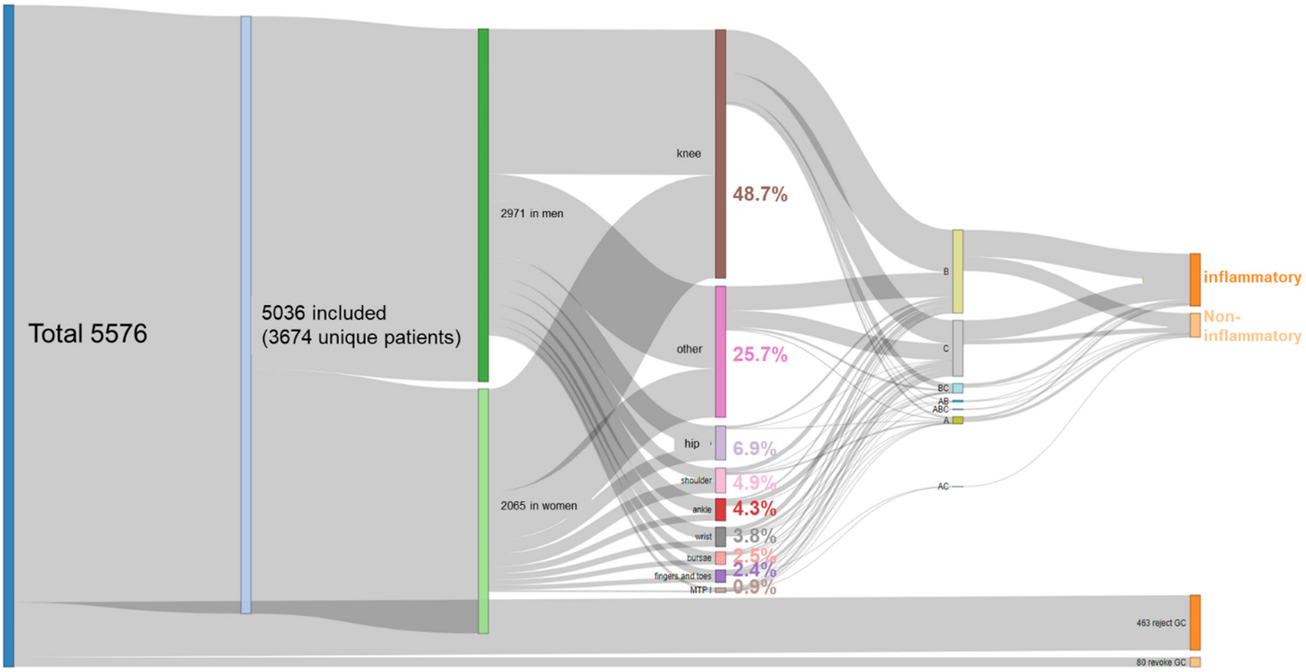

Supplementary Figure S1. Sankey plot indicating synovial fluid (SF) sample analysis algorithm and distribution according to subanalysis. Vertical columns from left to right: column 1 total size of screened samples; column 2 included samples with valid informed consent; column 3 sex distribution of samples; column 4 originating joints with relative percentages as indicated; column 5 crystal type with B = CPP, C = MSU, A = ARP, BC = CPP/MSU, AC = ARP/MSU, AB = ARP/CPP, ABC = ARP/CPP/MSU positive; column 6 inflammatory and norm/non-inflammatory cellular state as well samples with rejected or revoked general consent (GC).

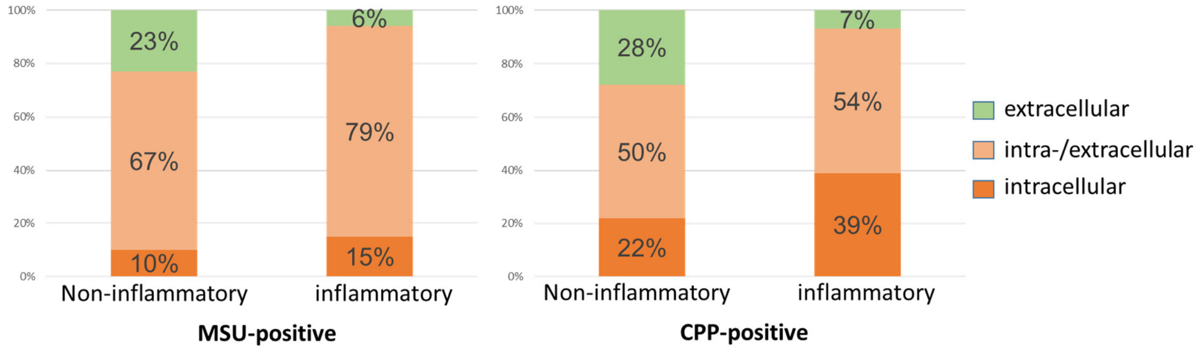

Supplementary Figure S2. Fractions of intracellular, intra-/extracellular and extracellular crystals according to qualitative evaluation by observers.
